# Complex Patterns of Gene Fission in the Eukaryotic Folate Biosynthesis Pathway

**DOI:** 10.1093/gbe/evu213

**Published:** 2014-09-23

**Authors:** Finlay Maguire, Fiona L. Henriquez, Guy Leonard, Joel B. Dacks, Matthew W. Brown, Thomas A. Richards

**Affiliations:** ^1^Department of Life Sciences, Natural History Museum, London, United Kingdom; ^2^Infection and Microbiology Research Group, Institute of Biomedical and Environmental Health Research, School of Science, University of the West of Scotland, Paisley, Renfrewshire, United Kingdom; ^3^Biosciences, University of Exeter, Geoffrey Pope Building, Exeter, United Kingdom; ^4^Department of Cell Biology, Faculty of Medicine and Dentistry, University of Alberta, Edmonton, Alberta, Canada; ^5^Department of Biological Sciences, Mississippi State University; ^6^Canadian Institute for Advanced Research, CIFAR Program in Integrated Microbial Biodiversity

**Keywords:** phylogenetics, comparative genomics, pterin biosynthesis, Diaphoretickes

## Abstract

Shared derived genomic characters can be useful for polarizing phylogenetic relationships, for example, gene fusions have been used to identify deep-branching relationships in the eukaryotes. Here, we report the evolutionary analysis of a three-gene fusion of *folB*, *folK*, and *folP,* which encode enzymes that catalyze consecutive steps in de novo folate biosynthesis. The *folK-folP* fusion was found across the eukaryotes and a sparse collection of prokaryotes. This suggests an ancient derivation with a number of gene losses in the eukaryotes potentially as a consequence of adaptation to heterotrophic lifestyles. In contrast, the *folB-folK-folP* gene is specific to a mosaic collection of Amorphea taxa (a group encompassing: Amoebozoa, Apusomonadida, Breviatea, and Opisthokonta). Next, we investigated the stability of this character. We identified numerous gene losses and a total of nine gene fission events, either by break up of an open reading frame (four events identified) or loss of a component domain (five events identified). This indicates that this three gene fusion is highly labile. These data are consistent with a growing body of data indicating gene fission events occur at high relative rates. Accounting for these sources of homoplasy, our data suggest that the *folB-folK-folP* gene fusion was present in the last common ancestor of Amoebozoa and Opisthokonta but absent in the Metazoa including the human genome. Comparative genomic data of these genes provides an important resource for designing therapeutic strategies targeting the de novo folate biosynthesis pathway of a variety of eukaryotic pathogens such as *Acanthamoeba castellanii*.

## Introduction

The resolution of ancient phylogenetic relationships is proving a difficult task (Philippe and Laurent 1998; Philippe 2000; Dagan and Martin 2006). Rare genomic characters such as: Insertions and/or deletions within open reading frames (ORFs), intron distribution, and gene fusions are potentially useful tools for polarizing evolutionary relationships and rooting trees (Jensen and Ahmad 1990; Philippe et al. 2000; Rokas and Holland 2000). In these cases, assuming parsimony, the logic proceeds that taxa A and B possess a rare genomic character, whereas taxa C and D do not, therefore taxa A and B are likely to be monophyletic to the exclusion of taxa C and D. The process of gene fusion and domain recombination is itself an important evolutionary process, leading to: Acquisition of new gene functions (Doolittle 1995), biochemical channeling (Miles et al. 1999), coregulation, colocalization, and potentially promoting the fixation of horizontally transferred genes (Andersson and Roger 2002; Yanai et al. 2002; Slot and Rokas 2010, 2011) see also (Lawrence and Roth 1996; Lawrence 1999; Walton 2000). The corollary with investigating gene fusions is that they are also subject to homoplasy in the form of: Horizontal gene transfer (HGT) (Andersson and Roger 2002; Yanai et al. 2002), separation (gene fission), gene duplication with differential loss of subsections of the gene (also a form of gene fission), total gene loss (Nakamura et al. 2007; Leonard and Richards 2012), or convergent evolution (Nara et al. 2000; Stover et al. 2005).

**Fig. 1.—:**
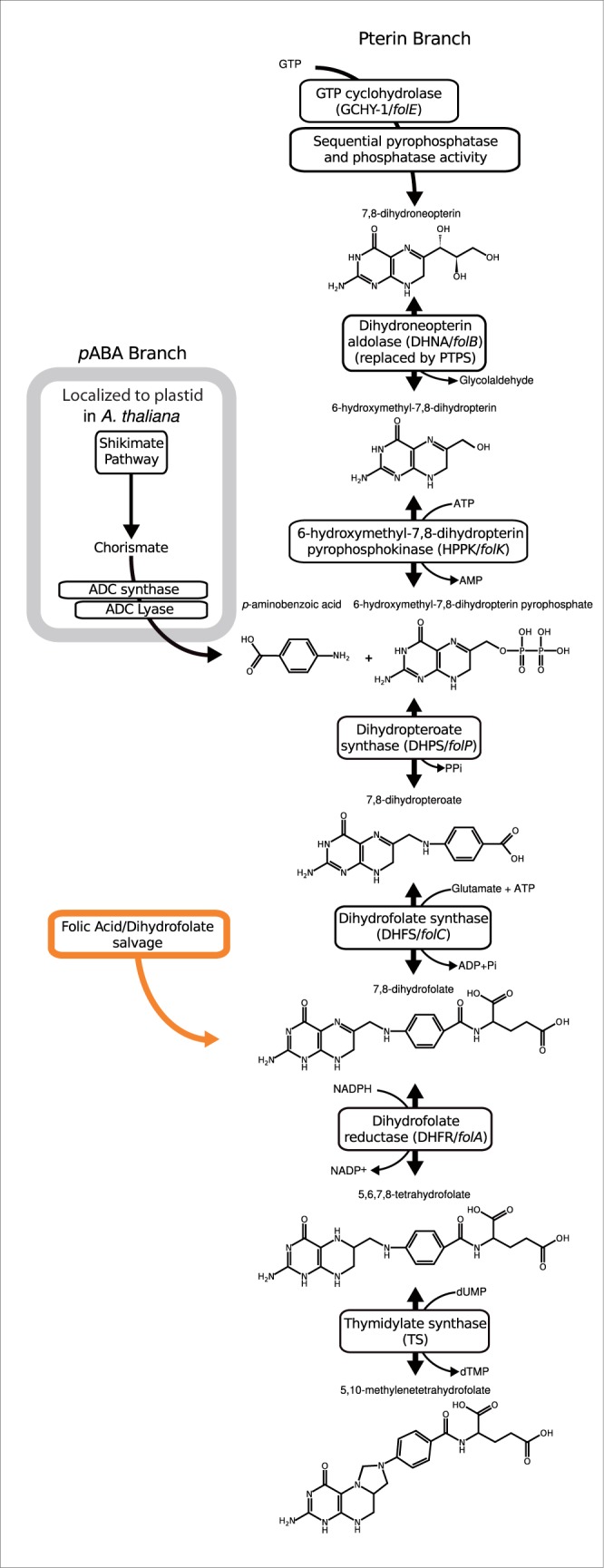
Part of the folate biosynthesis pathway with intermediate chemical states of the pathway illustrated. Protein and gene names that encode each step of the pathway are given.

**Fig. 2.—:**
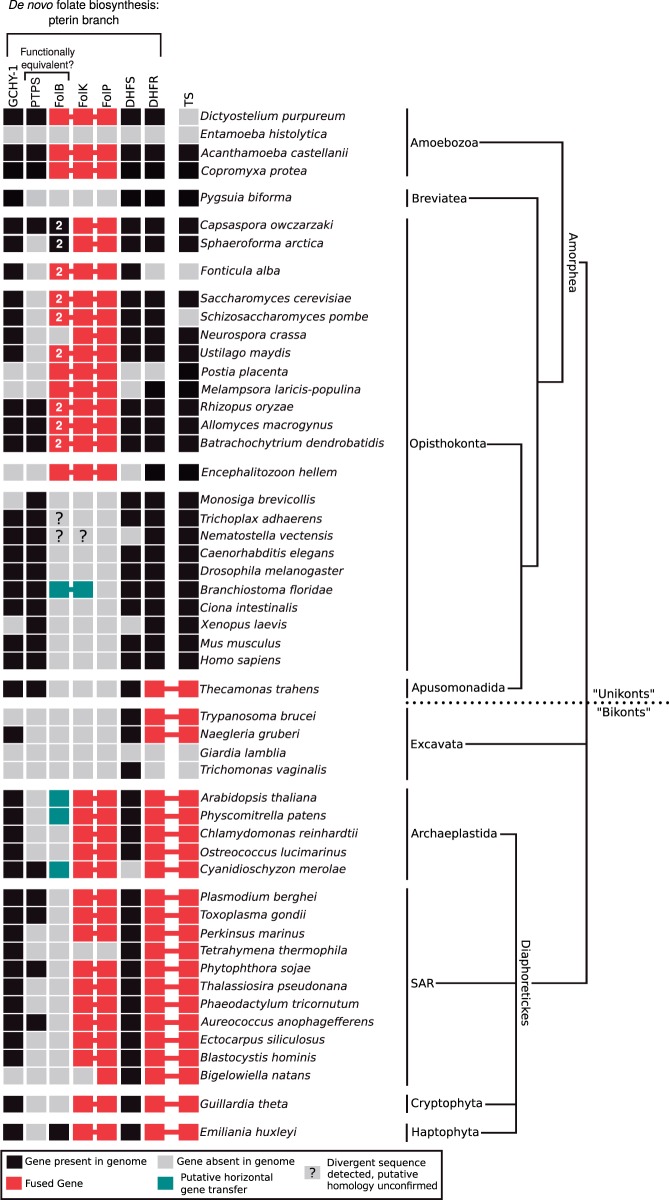
Presence, absence, and fusion state of putative folate pathway encoding genes across the eukaryotes. Taxonomic distribution of the pterin branch of the folate biosynthesis pathway. The red boxes and connecting lines indicate a gene fusion, black boxes represents presence of a putative homologue, and gray indicates gene not identified in the genome sequence data. Amoebozoa and Opisthokonta were formerly referred to as the “unikonts,” and likewise SAR, Excavata, and Archaeplastida were formerly referred to as the “bikonts.” Note that the putative *folB* of *Trichoplax adhaerens* and the putative *folB-folK* fusion of *Nematostella vectensis* were removed from phylogenetic analyses due to poor alignment of these sequences, as such their provenance and evolutionary ancestry remains questionable and are therefore indicated by a question mark at the relevant position.

Folate is an essential metabolite involved in the biosynthesis of: Adenine and thymidine bases, methionine and histidine amino acids, and formyl-tRNA (Brown 1971). Many plants protists, Fungi, Bacteria, and Archaea manufacture folate de novo (Cossins and Chen 1997; Levin et al. 2004; de Crecy-Lagard et al. 2007) principally via a double-branched pathway involving the pterin and pABA branches which feed into the step mediated by the enzyme encoded by *folP* (the pathway is illustrated in fig. 1 with gene and protein names listed). In the plant *Arabidopsis thaliana* many steps, including the proteins encoded by *folK-folP*, are localized to the mitochondria, whereas the enzymes that catalyze pABA synthesis are localized within the plastid organelle (de Crecy-Lagard et al. 2007). Folate salvage systems are also known from a range of taxa, where pterin and pABA-glutamate fragments produced by folate breakdown are fed into curtailed versions of the pathway (Orsomando et al. 2006; de Crecy-Lagard et al. 2007). For example, in some metazoans the core of the pathway is bypassed by folic acid uptake from food (Cossins 2000; Lucock 2000), leaving only the requirement for: Dihydrofolate reductase (DHFR) and thymidylate synthase (TS) (see figs. 1 and 2). Antifolate drugs (e.g., sulfonamides and sulfones) targeting the DHPS step in the pterin branch (encoded by *folP*) are therefore important antimicrobial agents (Lawrence et al. 2005) because host animals do not encode the equivalent metabolic trait. Additionally, drugs targeting the latter steps of the pathway (e.g., methotrexate which inhibits DHFR) are used in chemotherapy to target cancer cells (Huennekens 1994; Cossins and Chen 1997).

The genes that encode the folate biosynthesis enzymes DHFR and TS are fused in many eukaryotes (Stechmann and Cavalier-Smith 2002) resulting in synthesis of a two domain multifunctional protein. This character has been suggested to be an anciently derived synapomorphy uniting the “bikont” clade (Stechmann and Cavalier-Smith 2002, 2003), a group of “ancestrally biciliate eukaryotes” including the: Stramenopiles, Alveolata, Rhizaria (known collectively as the SAR supergroup), Excavata, Cryptophyta, Haptophyta, and Archaeplastida. However, several eukaryotic subgroups appear to have lost either the fused or unfused DHFR and TS-encoding genes (Simpson and Roger 2004; Roger and Simpson 2009) (fig. 2) making this an unreliable character for polarizing evolutionary relationships. In addition, the “bikont” grouping has been revised and these taxa, with the exception of the Excavata, are now grouped within Diaphoretickes (Adl et al. 2012). We also note that Cavalier-Smith has abandoned this rooting system (Cavalier-Smith 2010) in favor of a root within the Excavata (Simpson 2003) rendering the “bikonts” paraphyletic. Furthermore, although myosin II was thought to be exclusive to Amoebozoa and Opisthokonta taxa (Richards and Cavalier-Smith 2005) this gene architecture is found in Heterolobosea (Excavata) (Fritz-Laylin et al. 2010). This suggests a different or deeper ancestry of myosin II. Alternatively, this distribution pattern may be the result of HGT (Berney C, personal communication) with additional examples of HGT-derived genes shared by Heterolobosea and Amoebozoa (Andersson 2011; Herman et al. 2013) supporting the idea that HGT between these groups has played a role. However, an amended version of the “bikont” and “unikont” bifurcation recently gained some direct support using a rooted multigene phylogenetic analysis of genes derived through the mitochondrial endosymbiosis (Derelle and Lang 2012), but also see He et al. (2014) for an alternative tree topology derived from a similar analytical approach.

In 2005, Lawrence et al. published the structure of three components of the *Saccharomyces cerevisiae* folate biosynthesis pathway; a triple domain gene fusion, encompassing the DHNA, HPPK, and DHPS enzymes encoded by *folB*, *folK*, and *folP* genes—steps 3, 4, and 5 in pterin biosynthesis pathway (Lawrence et al. 2005) (fig. 1). Interestingly, gene fusions are common in secondary metabolic networks, for example, the shikimate pathway that forms the prerequisite to the pABA branch of folate biosynthesis is encoded by numerous variant gene fusions (Campbell et al. 2004; Richards et al. 2006) and genes which encode key enzymes of the pABA branch of folate biosynthesis are often found fused (de Crecy-Lagard et al. 2007). Here, we report a phylogenomic analysis of gene fusion characteristics in the pterin folate biosynthesis pathway across the eukaryotes. We use these data to investigate the evolutionary ancestry of the three-domain pterin biosynthesis gene fusion, identifying: a diversity of gene fusion architectures, gene fission events, and a number of gene losses. Using these results, we evaluate this three gene fusion character as synapomorphy for the monophyletic grouping of the Opisthokonta and Amoebozoa finding a high incidence of homoplasy.

## Materials and Methods

### *Cloning and Sequencing of Folate Triple Domain Gene Fusion from* Acanthamoeba castellanii *cDNA*

Using the partially assembled genome reads of the *Acanthamoeba castellanii* sequencing project (available at the Baylor College of Medicine—https://www.hgsc.bcm.edu/microbiome/acanthamoeba-castellani-neff, last accessed October 3, 2014), we designed a range of overlapping polymerase chain reaction (PCR) primers (Marshall 2004) to target different domain sections of the three folate biosynthetic genes *folB*, *folK*, and *folP* (see supplementary table S1, Supplementary Material online). *Acanthamoeba castellanii* Neff strain was grown axenically in a modified M11 defined media (Shukla et al. 1990) without folate (supplementary table S2, Supplementary Material online) to encourage the transcription of folate biosynthesis pathway genes. Cells were collected and suspended in 1 ml of trizol reagent (Invitrogen) and RNA extracted using the single-step acid guanidinium thiocyanate–phenol–chloroform protocol as described by Chomczynski and Sacchi (Chomczynski and Sacchi 1987). The cDNA was then synthesized using the AffinityScript kit with random hexamers (Stratagene). PCR amplification for target folate biosynthesis genes was conducted using Master Mix (Promega, containing 3 mM MgCl_2_, 400 µM of each dNTP, and 50 U/ml of Taq DNA polymerase) to create a 25 µl PCR reaction mix (12.5 µl of Master Mix), 1 µl each primer (10 µM), 9.5 µl of Milli-Q pure water (Millipore), and 1 µl of template cDNA). *Acanthamoeba* cDNA was diluted to approximately 100 ng/µl using spectrophotometery (NanoDrop ND-1000). Thermocycling followed an initial incubation at 95 °C for 5 min, and cycling conditions details in supplementary table S1, Supplementary Material online followed by a 72 °C–5 min elongation step. See supplementary table S1, Supplementary Material online, for details of PCR primers used. Successfully amplified PCR products were gel-purified (Wizard SV Gel and PCR Clean-Up kit, Promega) and cloned using TA-cloning (PCR StrataClone Cloning Kit, Agilent Technologies). Five clones were selected from each PCR reaction and externally sequenced using the M13/pUC vector primers via Sanger sequencing (Cogenic Beckman-Coulter sequencing service, High Wycombe). The flanking vector sequences were removed; the sequences trimmed to areas of high chromatograph quality and ambiguously defined bases corrected. The overlapping sequences were then assembled into contigs using Sequencher (Gene Codes) version 4.10.1 program (http://www.genecodes.com/) producing a high-confidence consensus sequence for a partial ORF for the *folB*, *folk,* and *folP* gene fusion (GenBank Acc: AFW17812.1). These data demonstrate that the *folB*, *folk,* and *folP* genes are transcribed as a single three-domain gene fusion. It should be noted that subsequently a draft genome and predicted proteome of *Acanthamoeba* has been released (Clarke et al. 2013), which contains the same gene fusion of near identical sequence (513/514 identities with no gaps—GenBank Acc: XP_004341460). The full-length gene derived from the genome sequence was used for the subsequent *folB, folk,* and *folP* phylogenetic analyses.

### Survey of Additional Protist Taxa Using RNA-Seq Data

We used the *Dictyostelium purpureum* (XP_003290941) *folB, folk*, and *folP* three gene fusion and *Bacillus cereus* single domain unfused-genes (*folB*—NP_829975.1, *folK*—ZP_03233543.1, *folP*—ZP_07056868.1) as a search query to identify putative homologues using the basic local alignment search tool (tBLASTn) against a set of protistan RNAseq “in-house” data sets. This data set included the unicellular opisthokont *Fonticula alba,* the amoebozoan *Copromyxa protea*, and the breviate *Pygsuia biforma* (PCbi66). From these data, we were able to identify components of the *folB, folk,* and *folP* genes from *Fonticula* and *Copromyxa*, but not in the breviate *P. biforma* (PCbi66). Phylogenomic analysis demonstrates that breviate flagellates are related to opisthokonts and the Apusomonadida (Brown et al. 2013).

For these RNAseq projects, total RNA was isolated using Tri-reagent (Sigma) following the protocol supplied by the manufacturer. Construction of cDNA libraries and Illumina RNAseq was performed by the Institut de Recherche en Immunologie et Cancérologie of Université de Montréal (Canada) for *Copromyxa protea* (strain CF08-5), the BROAD Institute (Boston) for *F. alba* (strain ATCC 38817), and Macrogen (South Korea) for the *P. biforma* (PCbi66). Raw sequence read data were filtered based on quality scores with the fastq_quality_filter program of FASTXTOOLS (http://hannonlab.cshl.edu/fastx_toolkit/), using a cutoff filter (a minimum 70% of bases must have quality of 20 or greater). Filtered sequences were then assembled into clusters using the Inchworm assembler of the TRINITY r2011-5-13 package (Grabherr et al. 2011). The *F. alba* assembly is available via the BROAD Institute; however, the other two assemblies are currently unreleased (manuscript in preparation). All unmasked protein alignments are included as supplementary material, Supplementary Material online, on GitHub (DOI: 10.5281/zenodo.11716) as MASE files which includes the alignment mask information (generated by Seaview [Galtier et al. 1996]).

### Comparative Genomics and Phylogenetic Analysis

Using BLASTp and tBLASTn (Altschul et al. 1990) we initially searched NCBI GenBank, the Joint Genome Institute (http://genome.jgi-psf.org/), and the Broad Institute (http://www.broadinstitute.org/) genome databases (as of November 2013) using three separate folate biosynthesis domains from *B. cereus* (*folB*—NP_829975.1, *folK*—ZP_03233543.1 and *folP*—ZP_07056868.1) and the *D. purpureum* (XP_003290941) *folB, folk,* and *folP* three gene fusion divided into the three-domain regions. Care was taken to survey the major eukaryotic, archaeal, and bacterial groups; to this end additional BLAST searches were conducted using multiple start seeds from diverse taxa to check for alternative sequence hits. The amino acid sequences gathered for each domain were run through the REFGEN tool (Leonard et al. 2009). The multiple sequence comparison by log-expectation program (v3.8.31) (Edgar 2004) was used to produce a multiple sequence alignment for each domain (*folB*, *folK* and *folP*). Alignments were then manually corrected and masked in SeaView (version 4.2.4) (Galtier et al. 1996). Sequences that caused an unacceptable loss of putatively informative sites (due to the sequence nonalignment or not masking well) or that formed long branches in preliminary analysis were removed. Duplicate entries from closely related taxa, for example, highly similar sequences from different representativeness of the same bacterial or fungal genus (e.g., *Escherichia*, *Bacillus,* and *Aspergillus*) or multiple highly similar genes from the same genome (sister branches on preliminary phylogenetic trees) were removed from the alignments.

Phylogenetic analysis was conducted using both Bayesian and maximum-likelihood methodologies with the model of amino acid substitution selected using ProtTest3 (version 3.2.1—[Darriba et al. 2011]—see supplementary figs. S1–S7, Supplementary Material online). Sequences shown to form long branches in the phylogenetic analysis were removed from the alignment to reduce the risk of long-branch attraction artifacts (Felsenstein 1978; Philippe 2000), for example, the Microsporidian: *Encephalitozoon hellem* ATCC 50504 *folB-folK-folP* gene fusion—XP_003887200, and *Plasmodium berghei folK-folP* gene fusion—XP_15149005 from the *folK* alignment, and the analyses rerun. The phylogenies were calculated using parallelized-PTHREADS RAxML (version 7.7—Stamatakis 2006) with 1,000 (nonrapid) bootstrap replicates and using the substitution matrix and gamma distribution identified using ProtTest3 (version 3.2.1) (Yang 1996; Darriba et al. 2011). In a subset of these analyses invariant sites were also included as a model parameter (in accordance with ProtTest3 recommendations), see the figure legends for supplementary figures S1–S7, Supplementary Material online, for more details of the models used. Bayesian phylogenies were also reconstructed using MrBayes (version 3.2). Each analysis was conducted as two independent runs of four metropolis-coupled Markov chain Monte Carlo [MCMCMC] chains and continued until convergence of these runs as determined using the Tracer (version 1.5) (Rambaut and Drummond 2007). Burn-in was then also determined using Tracer. The program TREENAMER (Leonard et al. 2009) was then run on the resulting tree files in order to restore the correct taxa names from the REFGEN tags used during phylogenetic processing. These analyses were also repeated using the same methods but focusing on a reduced taxon data set and a concatenation of the *folK* and *folP* alignments to tests for improved topology support for key nodes (supplementary figs. S4–S7, Supplementary Material online).

## Results

### Diversity of Gene Fusions in the Folate Biosynthesis Pathways

At the core of pterin branch of the folate biosynthesis pathway are three genes (*folB, folk,* and *folP*) that encode sequentially acting enzymes: DHNA*,* HPPK, and DHPS (fig. 1). In some fungi these are found as a single gene encoding a three-domain protein (e.g., *S. cerevisiae*: GenBank accession NP_014143.2—[Lawrence et al. 2005]) suggesting that gene fusion has played a role in the pterin branch of folate biosynthesis. To investigate the evolutionary ancestry of this gene fusion, we conducted comparative genomics of these three domains. These analyses demonstrated a discontinuous distribution across the eukaryotes suggesting a complex pattern of gene loss (fig. 2). We identified four different domain architectures, as defined by PFAM searches (Bateman et al. 2004), of the eukaryotic folate biosynthesis protein sequences sampled: 1) *folB-folB-folK-folP* found in a range of fungi and the opisthokont sorocarpic protist *F. alba*; 2) *folB-folK-folP* found in Amoebozoa, the basidiomycete fungi *Postia placenta, Coprinopsis cinerea,* and *Melampsora laricis-populina,* and the microsporidian *E. hellem*, (excluded from phylogenetic analysis because it formed a long branch in the phylogenies, like many other microsporidian sequences [Hirt et al. 1999]); 3) *folB-folK* found in two metazoans; and 4) *folK-folP* found in a subset of ascomycete fungi, *Puccinia graminis, Capsaspora owczarzaki*, *Sphaeroforma arctica*, and a diverse range of Diaphoretickes (fig. 2).

In many Diaphoretickes groups, including SAR, Cryptophyta, and the Excavata, we could not identify a *folB* gene using standard BLAST similarity searches (fig. 2). To confirm this result, we used a five iteration PSI-BLAST search using both the *B. cereus folB* gene and the *folB* domain of the *D. purpureum folB-folK-folP* gene fusion as a search seed against the NCBI GenBank nonredundant (NR) protein database (performed both as a general search and a search restricted to eukaryotic taxa). These analyses failed to identify any additional putative *folB* encoding genes in the eukaryotic genomes available in the GenBank NR database.

Pyruvoyltetrahydropterin synthase (PTPS) has been suggested to represent a functional replacement of the DHNA enzyme (*folB*) (Pribat et al. 2009). To investigate the possibility that this gene has functionally replaced *folB* in the Diaphoretickes and Excavata, or other eukaryotic groups, we searched the eukaryotes for the presence of genes with similar sequence characteristics across the genomes sampled (fig. 2). These analyses identified no clear pattern of PTPS/*folB* presence/absence, providing no support for this hypothesis that PTPS is acting as a like-for-like functional replacement of *folB* across the eukaryotes.

### Phylogenetic Analyses of the folB, folK, and folP Domains

To further investigate the evolutionary ancestry of the gene fusion character, we calculated individual phylogenies for the three pterin biosynthesis domains with both comprehensive and reduced taxa alignment sampling. The results of these phylogenies are shown in supplementary figures S1–S6, Supplementary Material online, with all six trees demonstrating low levels of topology support while many features of the eukaryotic sections of the tree topologies are inconsistent with established multigene phylogenetic trees (e.g., Rodriguez-Ezpeleta et al. 2005; Hampl et al. 2009; Derelle and Lang 2012; Torruella et al. 2012; Brown et al. 2013). This is typical of single-gene phylogenetic analysis using limited numbers of amino acid alignment characters (i.e., 78, 102, 175, 110, 102, 236 amino acid characters for supplementary figs. S1–S6, Supplementary Material online, respectively) and which encompasses ancient and divergent evolutionary groups. These alignment character numbers do not compare favourably to multigene analyses where it has been shown that in excess of 5,000 amino acid alignment characters are required to robustly resolve the Archaeplastida (Rodriguez-Ezpeleta et al. 2005). Although interestingly, Hampl et al. (2009) demonstrated that a low number of genes are sufficient to recover monophyly of the Opisthokonta branching sister to the Amoebozoa.

**Fig. 3.—:**
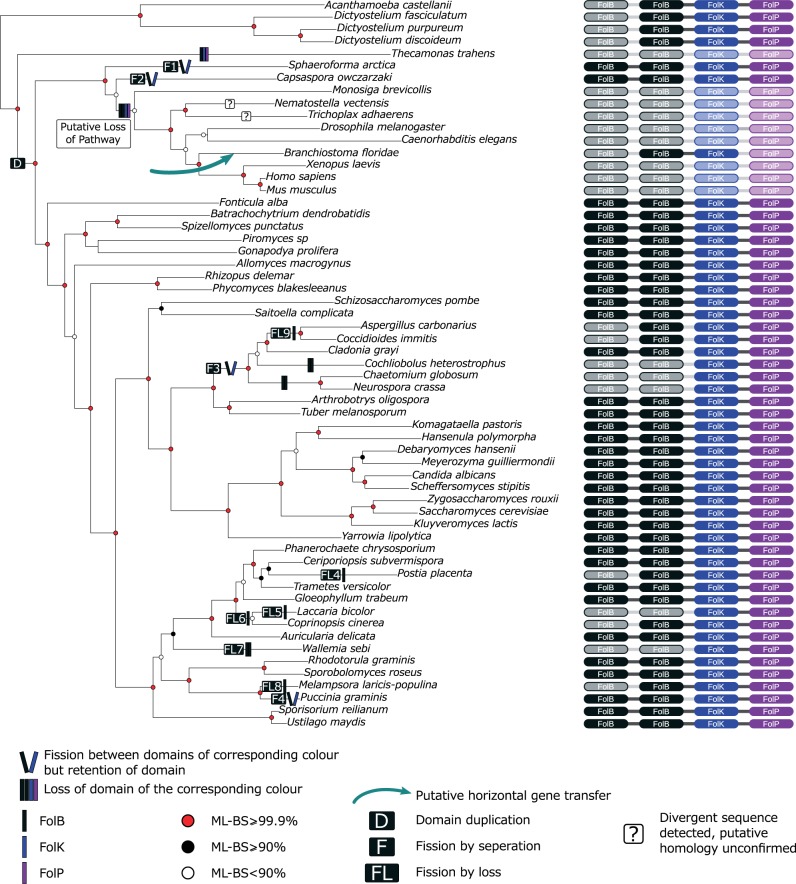
Phylogeny of the Apusomonadida, Breviata, Opisthokonta, and Amoebozoa demonstrating variation in the *folB-folK-folP* fusion gene. Tree topology was calculated using a concatenated alignment of conserved genes identified in (Torruella et al. 2012) and represents the best-known likelihood tree from 100 ML searches in RAxML (PROTCAT+LG) with 1,000 nonrapid bootstraps. ML-BS is an abbreviation of maximum likelihood bootstrap values, *FolB-folK-folP* fusion gene domain architecture of taxa included is listed down the right column, and fusion state is denoted by the presence/absence of connecting lines. Inferred gene/domain losses are shown as shadow domains. See key for guide to tree topology support values and character state changes. Domain duplication is indicated as (D) in a box of the appropriate domain colour, fission by domain loss events are denoted as (FL5–9) and specific fission events as (F1–4). Total losses of complete ORFs are not illustrated. Note that the putative *folB* of *Trichoplax adhaerens* and the putative *folB-folK* fusion of *Nematostella vectensis* were removed from phylogenetic analyses due to poor alignment of these sequences, as such their provenance and evolutionary ancestry remains questionable and are therefore indicated by a question mark at the relevant position.

Our analyses identified a *folB-folK* gene fusion in the metazoan *Branchiostoma floridae* genome assembly branching with a phylogenetic cluster of prokaryotes with moderate support within the comprehensive *folK* phylogeny (1/94% support for a grouping with *Planctomyces maris*—supplementary fig. S2, Supplementary Material online) and weak support in the reduced taxa *folK* analysis (0.939/27%—supplementary fig. S5, Supplementary Material online). The comprehensive *folB* phylogeny also shows the *Br. floridae folB-folK* gene fusion branching with prokaryote taxa with weak support (0.614/13%—supplementary fig. S1, Supplementary Material online). Collectively, these trees suggest that the *Br. floridae folB-folK* branching relationship is consistent with HGT into the *Br. floridae* genome or, alternatively, contamination of this genome project with a prokaryotic sequence. To explore these possibilities further, we found the genome sequence contig containing the *Br. floridae folB-folK* gene (GenBank acc: AC150408.2) demonstrating that the prokaryote like *Br. floridae folB-folK* gene is located in a 180,427 bp contig adjacent to genes that show standard patterns of animal sequence similarity. Analysis of the *B. belcheri* transcriptome demonstrated that an orthologue of the *Br. floridae folB-folK* gene is transcribed. Taken together these data suggest that the *Br. floridae folB-folK* gene is located on native source genome and it is not contamination. Therefore, it is likely to be a prokaryotic-derived HGT into this animal genome. However, it is interesting that an animal lineage could maintain only the first part of a pathway despite lacking the *folP* gene, whereas many other animal lineages have lost the entire pathway. Further to these data, we detected a putative *folB* gene in *Trichoplax adhaerens* and a putative *folB-folK* fusion gene in *Nematostella vectensis*. However, these genes were removed from further analyses due to difficulty in alignment of these sequences, as such their provenance and evolutionary ancestry remains questionable as noted on figures 2 and 3. These data suggest a partial folate biosynthesis pathway, or a pathway involving an alternative gene encoding the *folP* step present in *Branchiostoma*. Furthermore, we see evidence of incomplete pathways in other organisms, for example, the red alga *Cyanidioschyzon* lacks an identifiable standard *folP* gene (fig. 2)*.*

Monophyly of the three-domain gene fusion would signify that the *folB-folK* gene fusion was the product of a single evolutionary event. However, this relationship was not resolved with strong support in these analyses with only the *folB* phylogenies demonstrating a monophyletic grouping of the three domain *folB-folK-folP* gene fusions (both as *folB-folK-folP* and *folB-folB-folK-folP*) with weak topology support (i.e., 0.539/19% and 0.991/37% support [supplementary figs. S1 and S4, Supplementary Material online, respectively]). Importantly, we note that the only members of the Diaphoretickes and Excavata (formerly the “bikonts”) possessing a putative *folB* gene are the Archaeplastida and that the *folB* gene of this eukaryotic group branches separately from the other eukaryotes within a clade of bacterial genes with moderate-to-strong posterior probability/bootstrap support (supplementary fig. S1: 0.992/82%, Supplementary Material online and supplementary fig. S4: 1.000/94%, Supplementary Material online) suggesting a separate evolutionary ancestry of this gene to that of the Opisthokonta and Ameobozoa. Given the taxonomic distribution of the *folB* gene across the Archaeplastida (supplementary figs. S1 and S4, Supplementary Material online), this xenologue is most likely to have been derived either by an ancient horizontal gene transfer from a bacterial source into the Archaeplastida lineage or via the cyanobacterial endosymbiosis that gave rise to the plastid organelle, a process that has been suggested to lead to the acquisition of a number of genes of mixed bacterial ancestry (Brinkman et al. 2002; Martin et al. 2002). Using the *A. thaliana folB* gene, we searched for evidence of subcellular localization using the “cell eFP browser” (http://bar.utoronto.ca/cell_efp/cgi-bin/cell_efp.cgi?ncbi_gi=15229838, last accessed October 3, 2014) which suggested this gene product was localized to the cytosol or the mitochondria (supplementary table S3, Supplementary Material online). However, because the Archaeplastida *folB* is not an orthologue of the Opisthokonta/Amoebozoa version and no additional Diaphoretickes and Excavata *folB* orthologues are currently available, our *folB* phylogenetic analysis does not represent a strict test of the monophyly of the *folB-folK-folP* gene fusion within the eukaryotes.

Finally, in an attempt to improve tree resolution and to identify a resolved phylogeny, we conducted a concatenated phylogenetic analysis of the *folK* and *folP* genes (supplementary fig. S7, Supplementary Material online). This analysis again recovered a tree with low topology support values and taxonomic relationships inconsistent with established eukaryotic phylogenetic relationships (Rodriguez-Ezpeleta et al. 2005; Hampl et al. 2009; Derelle and Lang 2012; Torruella et al. 2012) and therefore provided no additional data to test the monophyly of *folB-folK-folP* three-domain gene fusions.

### folB Tandem Duplication in the Early Opisthokonta

Focusing on the “Opisthokonta and Amoebozoa *folB-folK-folP*” cluster, a clade specifically encompassing the *folB-folB-folK-folP* and *folB-folB* gene architectures found in Fungi, *F. alba, Sp. arctica,* and *C. owczarzaki* (fig. 2) forms with weak support in the reduced analysis (0.852/37%—supplementary fig. S4, Supplementary Material online). The taxon distribution of this character suggests that the *folB* tandem exon-duplication represents a novel genetic character that arose in the last common ancestor of the opisthokonts followed by the loss of these genes in Metazoa and some other opisthokont taxa (figs. 2 and 3). We can identify this pattern because multigene phylogenies place the *Sp. arctica* and *C. owczarzaki* branch sister to the choanoflagellates and metazoans (Torruella et al. 2012), so parsimoniously the *folB-folB* gene duplication predated the diversification of the major Opisthokonta clades (see fig. 3). The distribution of the Opisthokonta *folB* duplication therefore provides a character that infers the *folB-folK* fissions within the opisthokonts are nested events (see fig. 3—F1–4 fission events) and the ancestral Opisthokonta possessed a *folB-folK* gene fusion.

### Evidence of Gene Fission in the folB-folK-folP Gene Fusion

Our gene fusion character distribution analysis identifies nine fission events either by loss of one or two domains or by separation of the *folB-folB-folK-folP* fusion in the opisthokonts (fig. 3). Specifically, these events involve: Fission to form *folB-folB* and *folK-folP*, on the *Sp. arctica, C. owczarzaki,* and *Pu. graminis* branches (fig. 3, fission events F1, F2, and F4) and within the Pezizomycotina before the divergence of: *Aspergillus carbonarius, Coccidioides immitis, Cochliobolus heterostrophus, Cladonia grayi, Chaetomium globosum,* and *Neurospora crassa* (fig. 3, fission event F3). Furthermore, these data identify loss of one or both *folB* domains on five occasions in the branches leading to the basidiomycetes: *Co. cinerea*, *Laccaria bicolor*, *Wallemia sebi, Po. placenta,* and *M. laricis-populina* (fig. 3, fission by loss events, FL: 5–9) and the branch leading to the ascomycetes *As. carbonarius and Co. immitis*. In all nine cases, we reconfirmed the gene architectures by examining gene alignments and the synteny of each candidate fission gene in the relevant genome assemblies.

## Discussion

### Distribution of Putative Folate Biosynthesis Gene Homologues and Adaptation to Folate Heterotrophy

Using a comparative genomic and phylogenetic approach, we have identified the taxonomic distribution of a three protein domain encoding gene fusions in the pterin branch of the folate biosynthesis pathway. In the absence of strong phylogenetic signal demonstrating eukaryote-to-eukaryote HGT our analyses identified multiple gene loss events in different eukaryotic groups (e.g., Metazoa and Excavata), suggesting that the capacity to manufacture folate de novo has been lost on multiple occasions within the eukaryotes. This is consistent with adaptation of these lineages to acquiring folate or folate intermediates from food sources and/or host organisms. Specifically, the comparative genomic data demonstrate that a complete pterin branch is absent from the Metazoa sampled, consistent with the hypothesis that animals acquire folate using “intact folate salvage” from digested food (Lucock 2000), putatively maintaining the last two or three steps of the biosynthesis pathway to facilitate salvage of folic acid (figs. 1 and 2). A similar pattern of gene presence/absence was identified for the *Trypanosoma* (Excavata), *Naegleria* (Excavata), and *Thecamonas* (Apusomonadida) genomes, suggesting that these protists acquire folate, or precursors of folate (e.g., folic acid), by salvage from external sources. We can therefore infer that these heterotrophic characteristics have resulted in concordant loss of the de novo folate biosynthesis. Likewise the absence, or near absence, of the entire folate biosynthesis pathway in *Entamoeba, Trichomonas,* and *Giardia* suggests a dependence on hosts or phagocytosed food for provision of intact folate, as such inhibiting folate synthesis as a therapeutic target is not viable for these parasitic protists, but inhibition of uptake transporters of intact folate may offer an alternative therapeutic strategy.

In many Diaphoretickes genomes (e.g., taxa from the SAR group and Cryptophyta) both *folK* and *folP* genes were present, but a putative homologue of the *folB* gene was not identified. These results suggest that this part of the pathway is absent from these taxa or performed by a highly divergent or nonhomologous gene family. A paralogue of *folB*: *folX* has been identified in *Escherichia coli* with 30% identical amino acid residues. This protein was classified as an epimerase and performs the equivalent aldolase type reaction with less than 1% velocity as the DHNA encoded by the *Ec. coli folB* gene (Haussmann et al. 1998) suggesting this paralogue is not functionally equivalent. Comparative genomic analysis of the distribution of *folB* gene in prokaryotes identified many phylogenetically disparate groups without an identifiable putative homologue (de Crecy-Lagard et al. 2007) leading these authors to make two suggestions: 1) the enzyme that catalyses this step is encoded by a uncharacterized putative transaldolase gene often found to cluster in the same operons as *folK*, and/or 2) because other taxa lacked the *folB* gene and a putative alternative transaldolase-encoding gene; a currently unidentified gene family must encode this enzyme (de Crecy-Lagard et al. 2007). Later work then showed some evidence that the *folB* in many bacteria has been replaced with a functionally equivalent six-PTPS (Pribat et al. 2009). Analysis of eukaryotic genomes demonstrates many eukaryotic protists lacking an identifiable *folB* or PTPS encoding gene, suggesting that a currently unidentified functionally equivalent but phylogenetically dissimilar gene may encode an enzyme that catalyses this step.

### Gene Fusion as an Adaptation for Folate Biosynthesis

Our data identified a number of variant gene fusions in pterin branch of the folate biosynthesis genes. These included a gene consisting of three domains and therefore the likely product of two distinct gene fusion events. Our comparative genomic survey suggests that this characteristic is only found in opisthokont taxa including the: Fungi, *F. alba*, Microsporidia, and a range of Amoebozoa (e.g., *Dictyostelium, Acanthamoeba,* and *Copromyxa*). Moreover, two domain variations of these gene fusion forms were identified in a range of eukaryotes (fig. 2). Gene fusions have been identified elsewhere in the folate biosynthesis pathway (Stechmann and Cavalier-Smith 2002, 2003; de Crecy-Lagard et al. 2007) suggesting that gene fusion has been an important process in the evolution of the eukaryotic folate biosynthesis, possibly as a consequence of selection for: Cotranscription, colocalization, promotion of metabolic channeling, or a general improvement of enzyme kinetics (Welch and Gaertner 1975; Meek et al. 1985; Ivanetich and Santi 1990; Miles et al. 1999; Richards et al. 2006). This pattern is consistent with other secondary metabolic pathways that are also localized in the cytosol and show complex patterns of gene fusion (e.g., Nara et al. 2000; Stover et al. 2005; Richards et al. 2006).

A genome database search identified fragments of the *folB-folK-folP* genes in the *Ac. castellanii* sequencing project (Baylor College of Medicine—https://www.hgsc.bcm.edu/microbiome/acanthamoeba-castellani-neff, last accessed October 3, 2014) and within the recently completed genome sequence (Clarke et al. 2013). To confirm that this was a bona fide *folB-folK-folP* triple domain gene fusion, we performed nested PCR on cDNA derived from an axenic culture of *Ac. castellanii* Neff strain grown in folate-limiting conditions (GenBank Acc: AFW17812.1). This work confirmed that *Ac. castellanii* transcribes a single gene fusion encoding the *folB-folK-folP* domain architecture and provides evidence of active folate biosynthesis via a complete pterin branch in *Ac. castellanii. Acanthamoeba* can cause keratitis infection of the cornea linked to use of contaminated contact lenses (Radford et al. 1995). These data suggests the potential for antimicrobial agents that inhibit pterin branch of folate biosynthesis (e.g., sulfonamides and sulfones) as therapeutic treatment for *Acanthamoeba* keratitis or as an additive to eye-care and contact lens solutions to prevent infections. Exploiting metabolic differences between *Acanthamoeba* and the human host is a potentially important avenue to identify new antimicrobials and limit toxic effects (Roberts and Henriquez 2010), particularly in the eye. For example, sulphadiazine has been used to target different metabolic pathways for the successful inhibition of *Acanthamoeba* growth in vitro (Mehlotra and Shukla 1993) and encouraging reports of its use in vivo have been made in experimentally induced *Acanthamoeba* meningoencephalitis in mice (Rowan-Kelly et al. 1982) and in granulomatous amoebic encephalitis in AIDS patients (Seijo Martinez et al. 2000).

### Phylogenetic Evidence for Frequency of Gene Fusion and Fission Events

We conducted a series of phylogenetic analyses to investigate if the gene fusion characters were monophyletic and identify any cases of gene fissions. Our results demonstrate the presence of a complex pattern of gene loss (discussed above). Comparisons of the distribution of different folate fusion genes to the established Opisthokonta phylogeny (James et al. 2006; Torruella et al. 2012) combined with individual domain phylogenetic analyses suggest a minimum of nine gene fission events (five by fission through domain loss [deletion] and four by fission through separation and retention of a separate genes encoding the constituent domains) (fig. 3). These suggest that gene fissions occur at a high rate in this pathway and *folB-folK-folP* gene fusions are not stable characters. This is consistent with a growing body of data demonstrating that the process of gene/domain separation is an important factor in gene evolution (Kummerfeld and Teichmann 2005; Nakamura et al. 2007; Leonard and Richards 2012).

Next, we used phylogenetic analysis to polarize the ancestry of the *folB-folK-folP* gene fusion. Our phylogenetic analysis generally proved inconclusive, because we failed to recover tree resolution and specifically because there is no Diaphoretickes and Excavata orthologue of the Amoebozoa and Opisthokonta *folB* gene. Taken together the phylogenies, therefore, do not constitute an appropriate test of the monophyly of the three-domain gene fusion clade (i.e., Amoebozoa and Opisthokonta). Furthermore, as the individual folate pathway gene phylogenies were generally unresolved, it is possible that undetected cases of hidden paralogy, multiple *folB* tandem duplications, and HGT may have occurred in the evolution of this pathway. HGT is especially a concern as some literature suggests that gene clustering increases the possibility that genes become fixed by selection once they have undergone transfer. This is because they lead to the acquisition of functional modules, either as an operon and/or gene fusions (e.g., Andersson and Roger 2002; Slot and Rokas 2010, 2011). Such factors would therefore act to further complicate the evolution of this pathway, but at present are hard to quantify using single-gene phylogenies. As we saw no additional evidence for HGT other than that discussed (i.e., ancestral acquisition of the *folB* gene in the Archaeplastida and acquisition of a *folB-folK* gene fusion in *Branchiostoma* from a likely prokaryotic source), we use the more parsimonious interpretation of vertical inheritance to explain the gene distribution observed.

The phylogenies provided no strong support for the paraphyly and convergent evolution of the three-domain gene fusion in the Amoebozoa and Opisthokonta. Therefore, in the absence of strong signal to support an alternative hypothesis and based on current taxonomic distribution of this character, we currently favour the null hypothesis that the *folB-folK-folP* three-domain gene fusion is monophyletic and arose once and before the diversification of the opisthokonts and amoebozoans. We do acknowledge that alternative hypotheses involving fissions and loss in the Diaphoretickes and Excavata taxa, or convergent gene fusions in the Amoebozoa and Opisthokonta taxa are only slightly less parsimonious given current data. This is an important concern as our data demonstrated that this gene fusion is not a stable character, subject to frequent gene fission and partial and total gene loss. Consequently, perhaps the overriding message of this work is that rare-derived genomic characters, such as gene fusions, can be noisy and therefore these data should not be applied to resolving evolutionary relationships without testing their ancestry and susceptibility to homoplasy.

## Supplementary Material

Supplementary figures S1–S7 and tables S1–S4 are available at *Genome Biology and Evolution* online (http://www.gbe.oxfordjournals.org/).

Supplementary Data

## References

[evu213-B1] Adl SM (2012). The revised classification of eukaryotes. J Eukaryot Microbiol..

[evu213-B2] Altschul SF, Gish W, Miller W, Myers EW, Lipman DJ (1990). Basic local alignment search tool. J Mol Biol..

[evu213-B3] Andersson JO (2011). Evolution of patchily distributed proteins shared between eukaryotes and prokaryotes: *Dictyostelium* as a case study. J Mol Microbiol. Biotechnol..

[evu213-B4] Andersson JO, Roger AJ (2002). Evolutionary analyses of the small subunit of glutamate synthase: gene order conservation, gene fusions, and prokaryote-to-eukaryote lateral gene transfers. Eukaryot Cell..

[evu213-B5] Bateman A (2004). The Pfam protein families database. Nucleic Acids Res..

[evu213-B6] Brinkman FS (2002). Evidence that plant-like genes in *Chlamydia* species reflect an ancestral relationship between Chlamydiaceae, Cyanobacteria, and the chloroplast. Genome Res..

[evu213-B7] Brown GM (1971). The biosynthesis of pteridines. Adv Enzymol Relat Areas Mol Biol..

[evu213-B8] Brown MW (2013). Phylogenomics demonstrates that breviate flagellates are related to opisthokonts and apusomonads. Proc R Soc Lond B Biol Sci..

[evu213-B9] Campbell SA (2004). A complete shikimate pathway in *Toxoplasma gondii*: an ancient eukaryotic innovation. Int J Parasitol..

[evu213-B10] Cavalier-Smith T (2010). Kingdoms protozoa and chromista and the eozoan root of the eukaryotic tree. Biol Lett..

[evu213-B11] Chomczynski P, Sacchi N (1987). Single-step method of RNA isolation by acid guanidinium thiocyanate-phenol-chloroform extraction. Anal Biochem..

[evu213-B12] Clarke M (2013). Genome of *Acanthamoeba castellanii* highlights extensive lateral gene transfer and early evolution of tyrosine kinase signaling. Genome Biol..

[evu213-B13] Cossins EA (2000). The fascinating world of folate and one-carbon metabolism. Can J Bot..

[evu213-B14] Cossins EA, Chen L (1997). Folates and one-carbon metabolism in plants and fungi. Phytochemistry.

[evu213-B15] Dagan T, Martin W (2006). The tree of one percent. Genome Biol..

[evu213-B16] Darriba D, Taboada GL, Doallo R, Posada D (2011). ProtTest 3: fast selection of best-fit models of protein evolution. Bioinformatics.

[evu213-B17] de Crecy-Lagard V, El Yacoubi B, de la Garza RD, Noiriel A, Hanson AD (2007). Comparative genomics of bacterial and plant folate synthesis and salvage: predictions and validations. BMC Genomics.

[evu213-B18] Derelle R, Lang BF (2012). Rooting the eukaryotic tree with mitochondrial and bacterial proteins. Mol Biol Evol..

[evu213-B19] Doolittle RF (1995). The multiplicity of domains in proteins. Annu Rev Biochem..

[evu213-B20] Edgar RC (2004). MUSCLE: a multiple sequence alignment method with reduced time and space complexity. BMC Bioinformatics.

[evu213-B21] Felsenstein J (1978). Cases in which parsimony or compatibility methods will be positively misleading. Syst Zool..

[evu213-B22] Fritz-Laylin LK (2010). The genome of *Naegleria gruberi* illuminates early eukaryotic versatility. Cell.

[evu213-B23] Galtier N, Gouy M, Gautier C (1996). SEAVIEW and PHYLO_WIN: two graphic tools for sequence alignment and molecular phylogeny. Comput Appl Biosci..

[evu213-B24] Grabherr MG (2011). Full-length transcriptome assembly from RNA-Seq data without a reference genome. Nat Biotechnol..

[evu213-B25] Hampl V (2009). Phylogenomic analyses support the monophyly of Excavata and resolve relationships among eukaryotic “supergroups". Proc Natl Acad Sci U S A..

[evu213-B26] Haussmann C, Rohdich F, Schmidt E, Bacher A, Richter G (1998). Biosynthesis of pteridines in *Escherichia coli*. J Biol Chem..

[evu213-B27] He D (2014). An alternative root for the eukaryote tree of life. Curr Biol..

[evu213-B28] Herman EK (2013). The mitochondrial genome and a 60-kb nuclear DNA segment from *Naegleria fowleri*, the causative agent of primary amoebic meningoencephalitis. J Eukaryot Microbiol..

[evu213-B29] Hirt RP (1999). Microsporidia are related to Fungi: evidence from the largest subunit of RNA polymerase II and other proteins. Proc Natl Acad Sci. U S A..

[evu213-B30] Huennekens FM (1994). The methotrexate story: a paradigm for development of cancer chemotherapeutic agents. Adv Enzyme Regul..

[evu213-B31] Ivanetich KM, Santi DV (1990). Bifunctional thymidylate synthase-dihydrofolate reductase in protozoa. FASEB J..

[evu213-B32] James TY (2006). Reconstructing the early evolution of Fungi using a six-gene phylogeny. Nature.

[evu213-B33] Jensen RA, Ahmad S (1990). Nested gene fusion markers of phylogenetic branch points in prokaryotes. Trends Ecol Evol..

[evu213-B34] Kummerfeld SK, Teichmann SA (2005). Relative rates of gene fusion and fission in multi-domain proteins. Trends Genet..

[evu213-B35] Lawrence J (1999). Selfish operons: the evolutionary impact of gene clustering in prokaryotes and eukaryotes. Curr Opin Genet Dev..

[evu213-B36] Lawrence JG, Roth JR (1996). Selfish operons: horizontal transfer may drive the evolution of gene clusters. Genetics.

[evu213-B37] Lawrence MC (2005). The three-dimensional structure of the bifunctional 6-hydroxymethyl-7,8-dihydropterin pyrophosphokinase/dihydropteroate synthase of *Saccharomyces cerevisiae*. J Mol Biol..

[evu213-B38] Leonard G, Richards TA (2012). Genome-scale comparative analysis of gene fusions, gene fissions, and the fungal tree of life. Proc Natl Acad Sci U S A..

[evu213-B39] Leonard G, Stevens JR, Richards TA (2009). REFGEN and TREENAMER: automated sequence data handling for phylogenetic analysis in the genomic era. Evol Bioinform Online.

[evu213-B40] Levin I, Giladi M, Altman-Price N, Ortenberg R, Mevarech M (2004). An alternative pathway for reduced folate biosynthesis in bacteria and halophilic archaea. Mol Microbiol..

[evu213-B41] Lucock M (2000). Folic acid: nutritional biochemistry, molecular biology, and role in disease processes. Mol Genet Metab..

[evu213-B42] Marshall OJ (2004). PerlPrimer: cross-platform, graphical primer design forstandard, bisulphite and real-time PCR. Bioinformatics.

[evu213-B43] Martin W (2002). Evolutionary analysis of *Arabidopsis*, cyanobacterial, and chloroplast genomes reveals plastid phylogeny and thousands of cyanobacterial genes in the nucleus. Proc Natl Acad Sci U S A..

[evu213-B44] Meek TD, Garvey EP, Santi DV (1985). Purification and characterization ofthe bifunctional thymidylate synthetase-dihydrofolate reductase from methotrexate-resistant *Leishmania tropica*. Biochemistry.

[evu213-B45] Mehlotra RK, Shukla OP (1993). In vitro susceptibility of *Acanthamoeba culbertsoni* to inhibitors of folate biosynthesis. J Eukaryot Microbiol..

[evu213-B46] Miles EW, Rhee S, Davies DR (1999). The molecular basis of substrate channeling. J Biol Chem..

[evu213-B47] Nakamura Y, Itoh T, Martin W (2007). Rate and polarity of gene fusion and fission in *Oryza sativa* and *Arabidopsis thaliana*. Mol Biol Evol..

[evu213-B48] Nara T, Hashimoto T, Aoki T (2000). Evolutionary implications of the mosaic pyrimidine-biosynthetic pathway in eukaryotes. Gene.

[evu213-B49] Orsomando G (2006). Evidence for folate-salvage reactions in plants. Plant J..

[evu213-B51] Philippe H (2000). Opinion: long branch attraction and protist phylogeny. Protist.

[evu213-B52] Philippe H (2000). Early-branching or fast-evolving eukaryotes? An answer based on slowly evolving positions. Proc R Soc Lond B Biol Sci..

[evu213-B54] Philippe H, Laurent J (1998). How good are deep phylogenetic trees?. Curr Opin Genet Dev..

[evu213-B55] Pribat A (2009). 6-pyruvoyltetrahydropterin synthase paralogs replace the folate synthesis enzyme dihydroneopterin aldolase in diverse bacteria. J Bacteriol..

[evu213-B56] Radford CF, Bacon AS, Dart JK, Minassian DC (1995). Risk factors for *Acanthamoeba* keratitis in contact lens users: a case-control study. BMJ..

[evu213-B57] Rambaut A, Suchard MA, Xie D, Drummond AJ (2014). Tracer v1.6. [cited 2014 Oct 3]. http://beast.bio.ed.ac.uk/Tracer.

[evu213-B58] Richards TA, Cavalier-Smith T (2005). Myosin domain evolution and the primary divergence of eukaryotes. Nature.

[evu213-B59] Richards TA (2006). Evolutionary origins of the eukaryotic shikimate pathway: gene fusions, horizontal gene transfer, and endosymbiotic replacements. Eukaryot Cell..

[evu213-B60] Roberts CW, Henriquez FL (2010). Drug target identification, validation, characterisation and exploitation for treatment of *Acanthamoeba* (species) infections. Exp Parasitol..

[evu213-B61] Rodriguez-Ezpeleta N (2005). Monophyly of primary photosynthetic eukaryotes: green plants, red algae, and glaucophytes. Curr Biol..

[evu213-B62] Roger AJ, Simpson AG (2009). Evolution: revisiting the root of the eukaryote tree. Curr Biol..

[evu213-B63] Rokas A, Holland PWH (2000). Rare genomic changes as a tool for phylogenetics. Trends Ecol Evol..

[evu213-B64] Rowan-Kelly B, Ferrante A, Thong YH (1982). The chemotherapeutic value of sulphadiazine in treatment of *Acanthamoeba* meningoencephalitis in mice. Trans R Soc Trop Med Hyg..

[evu213-B65] Seijo Martinez M (2000). Granulomatous amebic encephalitis in a patient with AIDS: isolation of *Acanthamoeba* sp. group II from brain tissue and successful treatment with sulfadiazine and fluconazole. J Clin Microbiol..

[evu213-B66] Shukla OP, Kaul SM, Mehlotra RK (1990). Nutritional studies on *Acanthamoeba culbertsoni* and development of chemically defined medium. J Protozool..

[evu213-B67] Simpson AG (2003). Cytoskeletal organization, phylogenetic affinities and systematics in the contentious taxon Excavata (Eukaryota). Int J Syst Evol Microbiol..

[evu213-B68] Simpson AG, Roger AJ (2004). The real ‘kingdoms' of eukaryotes. Curr Biol..

[evu213-B69] Slot JC, Rokas A (2010). Multiple GAL pathway gene clusters evolved independently and by different mechanisms in fungi. Proc Natl Acad Sci U S A..

[evu213-B70] Slot JC, Rokas A (2011). Horizontal transfer of a large and highly toxic secondary metabolic gene cluster between fungi. Curr Biol..

[evu213-B50] Stamatakis A (2006). RAxML-VI-HPC: maximum likelihood-based phylogenetic analyses with thousands of taxa and mixed models. Bioinformatics.

[evu213-B71] Stechmann A, Cavalier-Smith T (2002). Rooting the eukaryote tree by using a derived gene fusion. Science.

[evu213-B72] Stechmann A, Cavalier-Smith T (2003). The root of the eukaryote tree pinpointed. Curr Biol..

[evu213-B73] Stover NA, Cavalcanti AR, Li AJ, Richardson BC, Landweber LF (2005). Reciprocal fusions of two genes in the formaldehyde detoxification pathway in ciliates and diatoms. Mol Biol Evol..

[evu213-B74] Torruella G (2012). Phylogenetic relationships within the opisthokonta based on phylogenomic analyses of conserved single-copy protein domains. Mol Biol Evol..

[evu213-B75] Walton JD (2000). Horizontal gene transfer and the evolution of secondary metabolite gene clusters in fungi: an hypothesis. Fungal Genet Biol..

[evu213-B76] Welch GR, Gaertner FH (1975). Influence of an aggregated multienzyme system on transient time: kinetic evidence for compartmentation by an aromatic-amino-acid synthesizing complex of *Neurospora crassa*. Proc Natl Acad Sci U S A..

[evu213-B77] Yanai I, Wolf YI, Koonin EV (2002). Evolution of gene fusions: horizontal transfer versus independent events. Genome Biol..

[evu213-B78] Yang Z (1996). Among-site rate variation and its impact on phylogenetic analyses. Trends Ecol Evol..

