# Supplementary material for: Complex Patterns of Gene Fission in the Eukaryotic Folate Biosynthesis Pathway
Source: Genome Biol Evol. 2014 Sep 23;6(10):2709–20. doi: 10.1093/gbe/evu213 (PMC4224340; doi:10.1093/gbe/evu213)
Supplement: Supplementary Data [file supp_6_10_2709__index.html]

Complex Patterns of Gene Fission in the Eukaryotic Folate Biosynthesis Pathway — Supplementary Data 

# Complex Patterns of Gene Fission in the Eukaryotic Folate Biosynthesis Pathway

## Supplementary Data

files

**Files in this Data Supplement:**

- Supplementary Data - zip file
