# Supplementary material for: Complex Patterns of Gene Fission in the Eukaryotic Folate Biosynthesis Pathway
Source: Genome Biol Evol. 2014 Sep 23;6(10):2709–20. doi: 10.1093/gbe/evu213 (PMC4224340; doi:10.1093/gbe/evu213)
Supplement: Supplementary Data [file supp_evu213_suppl_data.zip › Maguire_et_al_Table S1-S3.docx]

**Table S1:** PCR primers and thermo-cycling conditions.

| **FWD Primer** | **Sequence (5’-3’)** | **REV Primer** | **Sequence(5’-3’)** | **Thermo-cycling conditions** |
| --- | --- | --- | --- | --- |
| FOLB18F | GGATCTGATGGTGCAGGCCATCC | FOLP1662R | CCGCCAGCTGAGCCGAATCG | Cycles: 20  Denaturing: 60 s @ 95 ^o^c  Annealing: 40 s @ 60 ^o^c  Elongation: 100 s @72 ^o^c |
| FOLB168F | CAAGGCCGTCGTGGCCTACA | FOLP1662R | CCGCCAGCTGAGCCGAATCG | Cycles: 20  Denaturing: 60 s @ 95 ^o^c  Annealing: 40 s @ 60 ^o^c  Elongation: 100 s @72 ^o^c |
| FOLB168F | CAAGGCCGTCGTGGCCTACA | FOLK620R | AGAGGGCCGAGGTGCTGATGATGT | Cycles: 20  Denaturing: 60 s @ 95 ^o^c  Annealing: 40 s @ 61 ^o^c  Elongation: 40 s @ 72 ^o^c |
| FOLK623F | TCATCAGCACCTCGGCCCTCTACC | FOLP1151R | GGGCGTCGCGTTGATGATGC | Cycles: 20  Denaturing: 60 s @ 95 ^o^c  Annealing: 40 s @ 62 ^o^c  Elongation: 40 s @ 72 ^o^c |

**Table S2:** Modified M11 defined media (Shukla, et al. 1990) used to culture *Acanthamoeba.*

| **Amino acids** | **Conc.(mg/litre)** |
| --- | --- |
| L – Arginine | 825 |
| L – Methionine | 300 |
| L – Leucine | 900 |
| L – Isoleucine | 600 |
| L – Valine | 700 |
| Glycine | 1500 |
| L - Lysine. HCl | 1250 |
| L – threonine | 500 |
| **Salts** | **Conc. (mg/litre)** |
| MgSO_4_.7H_2_O | 985 |
| CaCl_2_.2H_2_O | 58.8 |
| (NH_4_)_2_SO_4_FeSO_4_.6H_2_O | 19.6 |
| Na_2_HPO_4_.2H_2_O | 445 |
| KH_2_PO_4_ | 340 |
| Na Citrate | 1000 |
| **Carbohydrates** | **Conc. (mg/litre)** |
| Glucose | 36000 |
| **Trace elements** | **Conc. (mg/litre)** |
| ZnSO_4_.7H_2_O | 1 |
| MnCl_2_.4H_2_O | 2.3 |
| (NH_4_)_6_Mo_7_O_24_.4H_2_O | 0.4 |
| CoCl_2_ | 0.017 |
| CuSO_4_.5H_2_O | 0.0033 |
| H_3_BO_3_ | 0.1 |
| EDTA | 0.01 |
| **Vitamins** | **Conc. (mg/litre)** |
| Biotin | 0.25 |
| B12 | 0.00125 |
| Thiamine HCl | 1.25 |

(All reagents obtained from: Sigma-Aldrich)

**Table S3:** TargetP and Predotar localisation survey of photosynthetic Diaphoretickes taxa

|  | ***FolB (TargetP, Predotar)*** | ***FolK-P (TargetP, Predotar)*** |
| --- | --- | --- |
| ***Guillardia theta*** | NA, NA, | M, _, |
| ***Arabidopsis thaliana*** | S, _, | M, M, |
| ***Physcomitrella patens*** | _, _, | _, _, |
| ***Cyanidioschyzon merolae*** | M, ER, | _, _, |
| ***Thalassiosira pseudonana*** | NA, NA, | M, _, |
| ***Ectocarpus siliculosus*** | NA, NA, | M, M, |
| ***Emiliania huxleyi*** | NA, NA, | M, M, |
| ***Toxoplasma gondii (PATS)*** | NA, NA, | M, ER |

M = Mitochondrial, S = Secretory, ER = Endoplasmic Reticulum, NA = No detected signal motifs

**Table S4 (supplementary file):** a list of all genome projects screened using a primary BLAST search to recover candidate *folB, folK* and *folP* genes.
