# Supplementary figures and images for: Complex Patterns of Gene Fission in the Eukaryotic Folate Biosynthesis Pathway
Source: Genome Biol Evol. 2014 Sep 23;6(10):2709–20. doi: 10.1093/gbe/evu213 (PMC4224340; doi:10.1093/gbe/evu213)

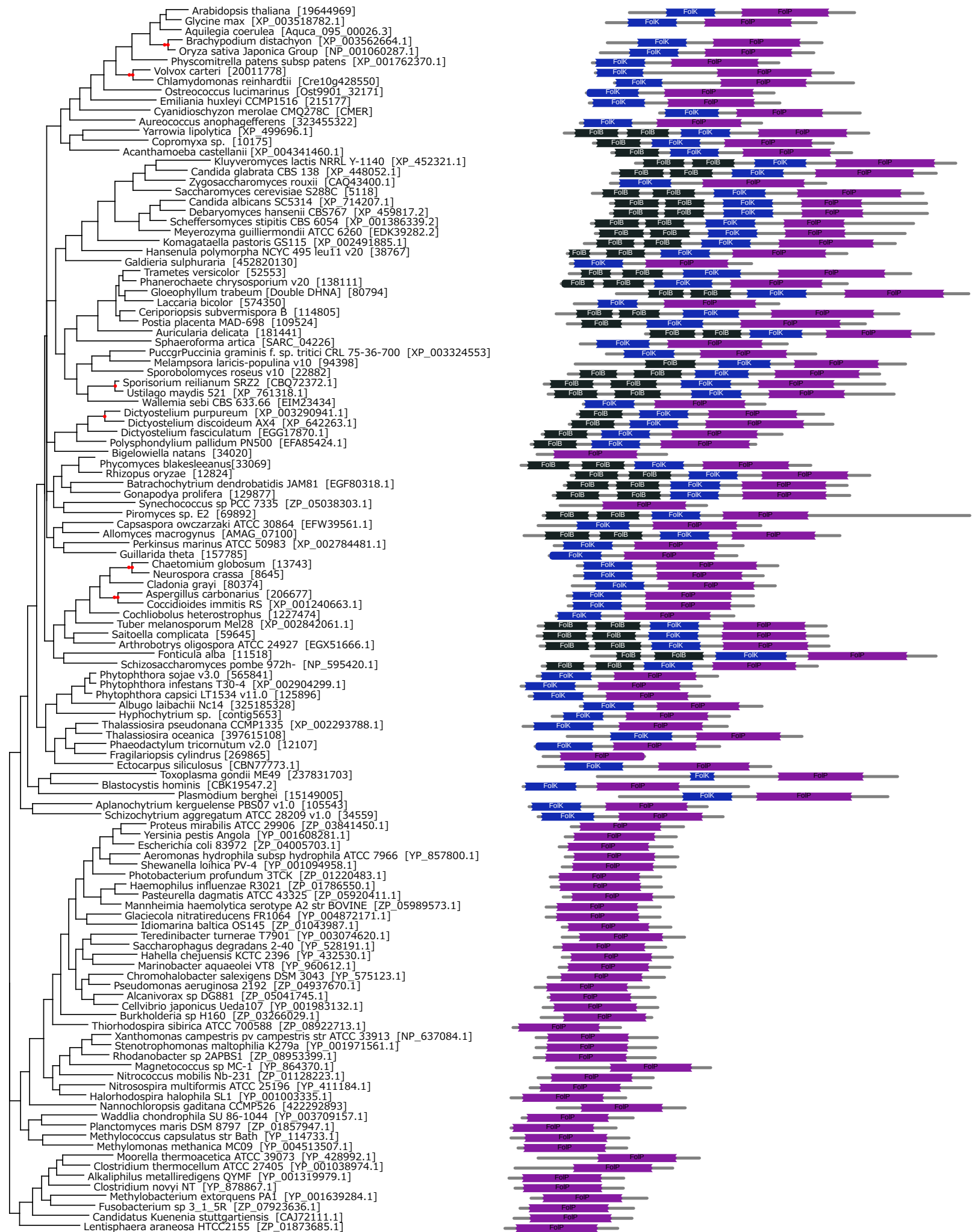

• BPP>0.95  
 • BPP>0.95 and ML-BS>75%  
 • BPP>0.95 and ML-BS<75%

Supplement: Supplementary Data [file supp_evu213_suppl_data.zip › S3_FolP_full_mb_raxml.pdf]

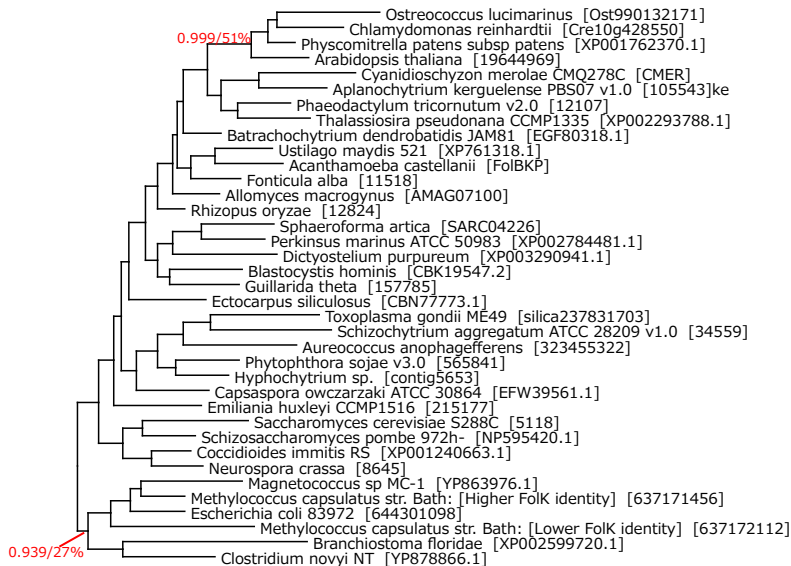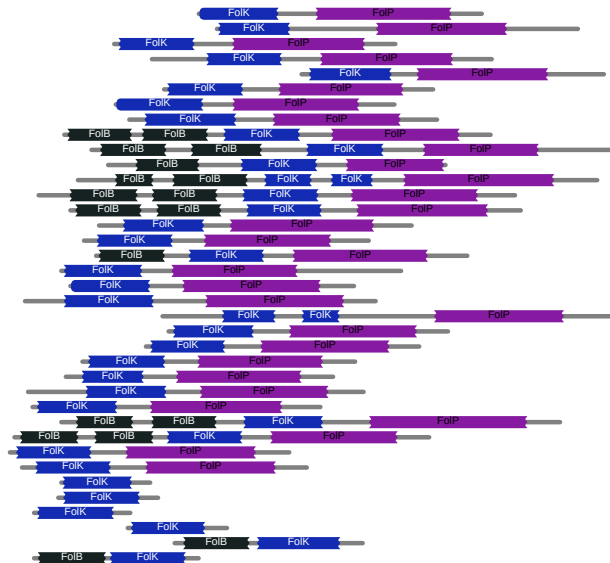

Maguire et al., Figure S5

Supplement: Supplementary Data [file supp_evu213_suppl_data.zip › S5_FolK_reduced_mb_raxml.pdf]

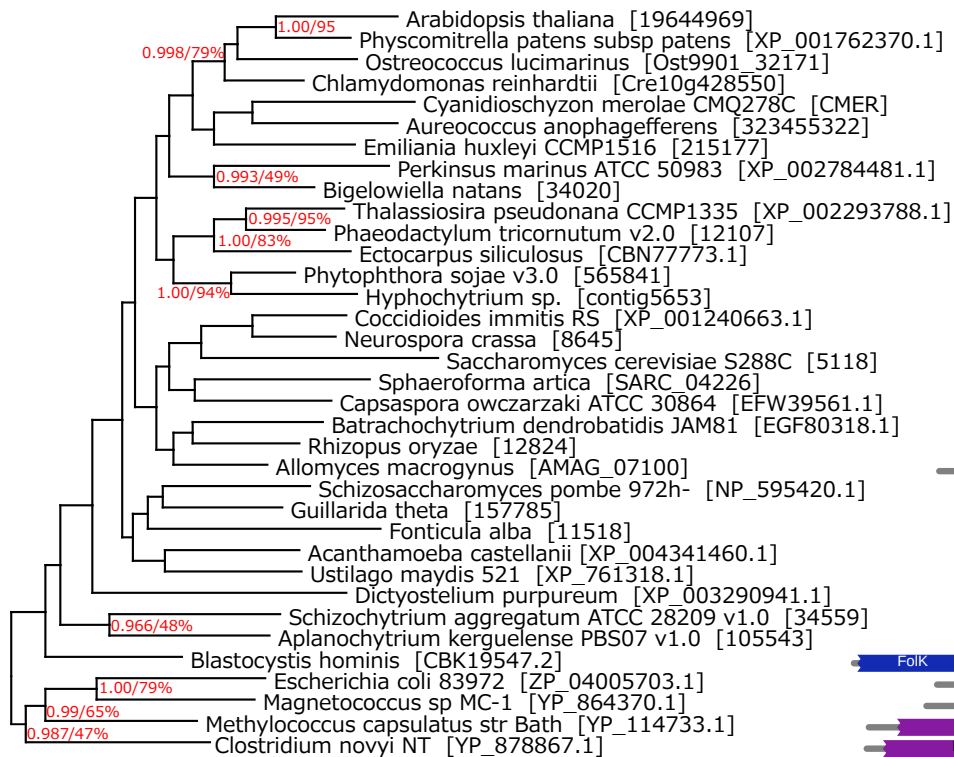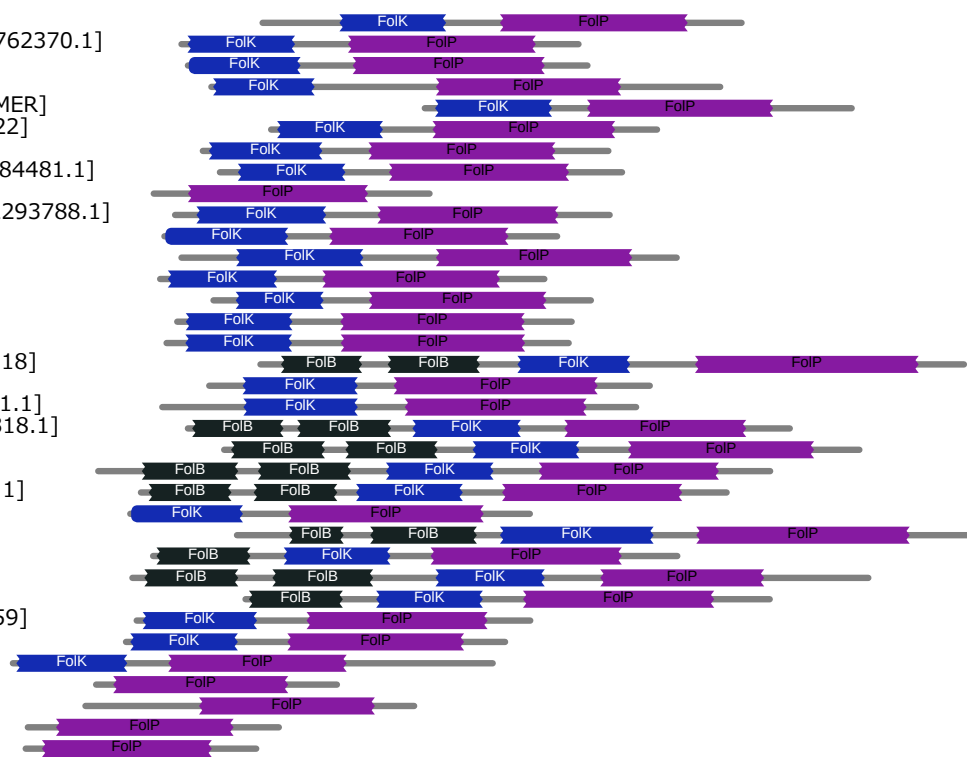

Maguire et al., Figure S6

Supplement: Supplementary Data [file supp_evu213_suppl_data.zip › S6_FolP_reduced_mb_raxml.pdf]

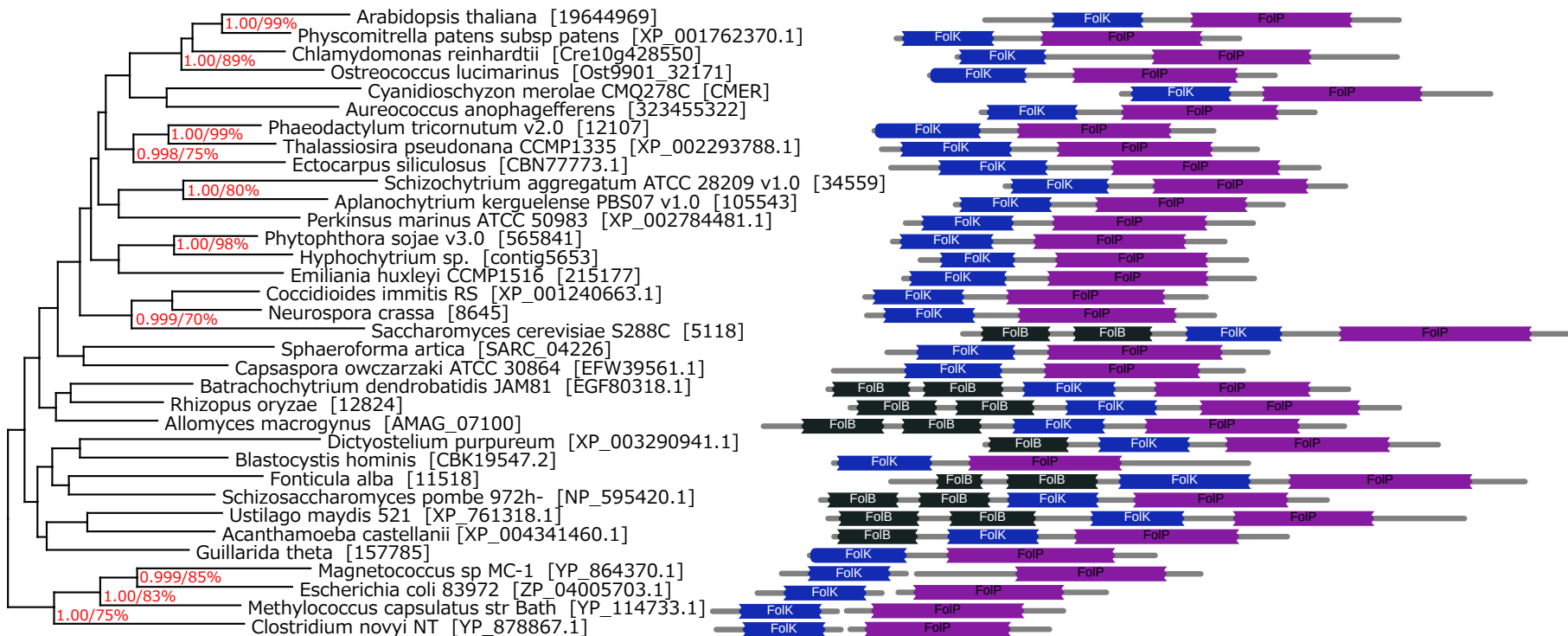

Maguire et al., Figure S7

Supplement: Supplementary Data [file supp_evu213_suppl_data.zip › S7_FolKP_mb_raxml.pdf]

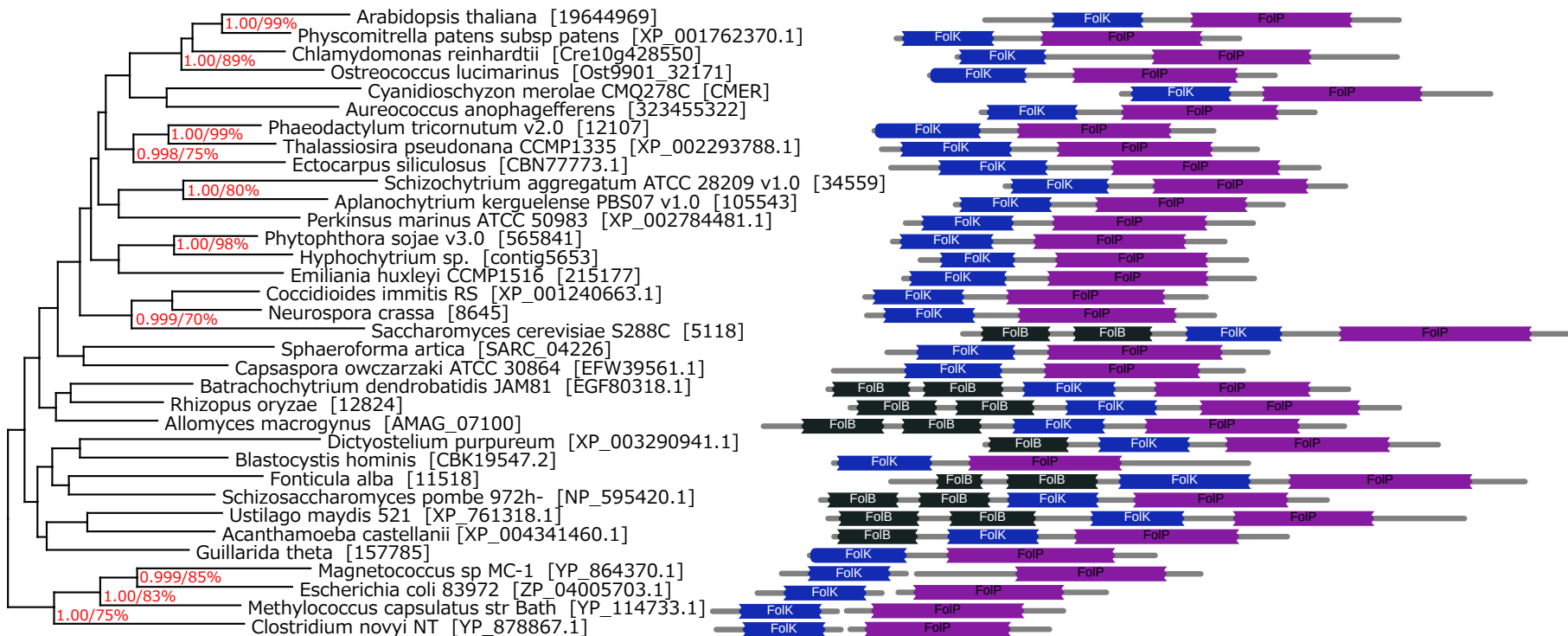

Maguire et al., Figure S7

Supplement: Supplementary Data [file supp_evu213_suppl_data.zip › S1_FolB_full_mb_raxml.pdf]

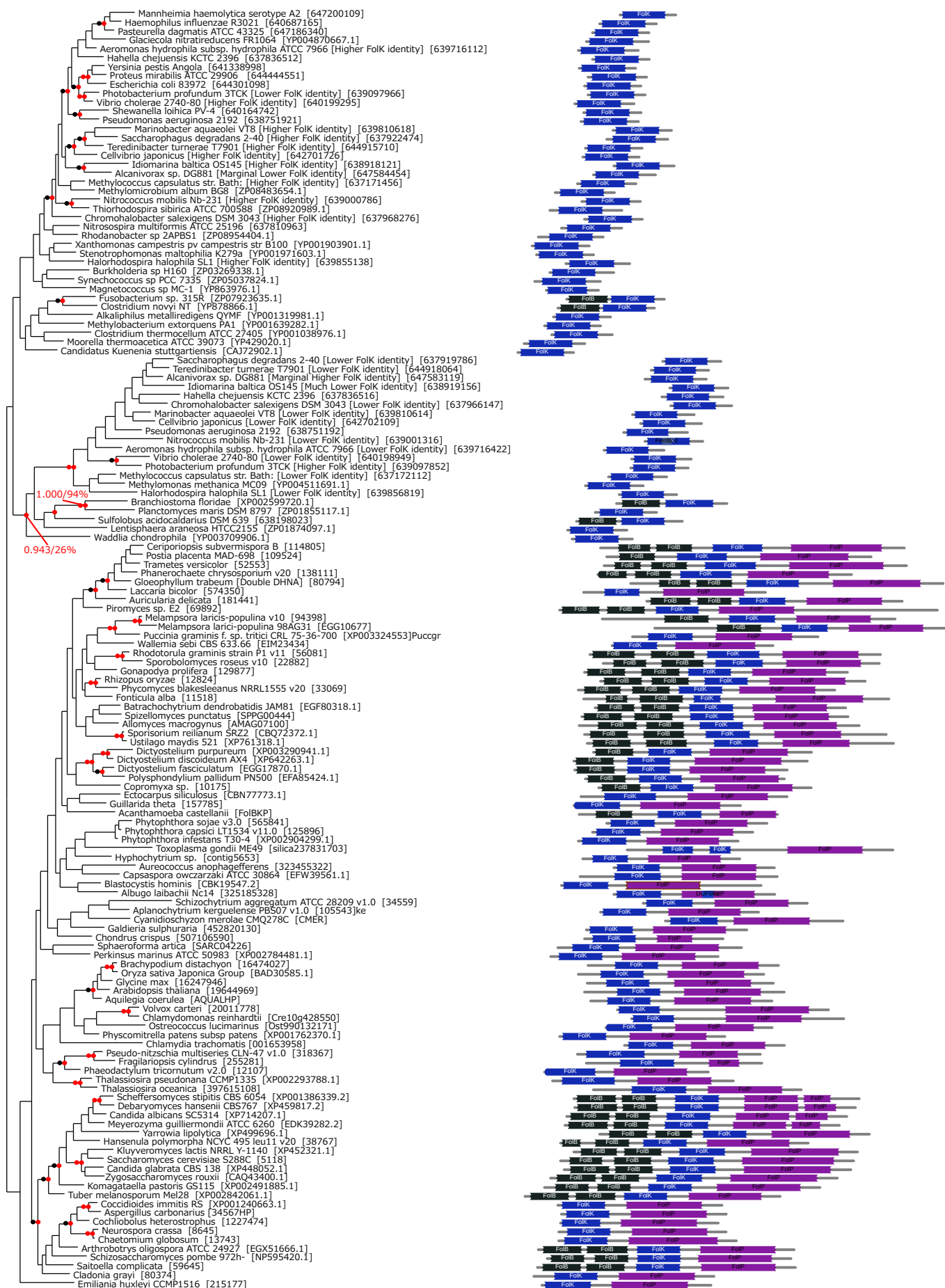

• BPP>0.95  
 • BPP>0.95 and ML-BS>75%  
 • BPP>0.95 and ML-BS<75%

Supplement: Supplementary Data [file supp_evu213_suppl_data.zip › S2_FolK_full_mb_raxml.pdf]
