# Supplementary material for: Complex Patterns of Gene Fission in the Eukaryotic Folate Biosynthesis Pathway
Source: Genome Biol Evol. 2014 Sep 23;6(10):2709–20. doi: 10.1093/gbe/evu213 (PMC4224340; doi:10.1093/gbe/evu213)
Supplement: Supplementary Data [file S4_FolB_reduced_mb_raxml.pdf › S4_FolB_reduced_mb_raxml.pdf]

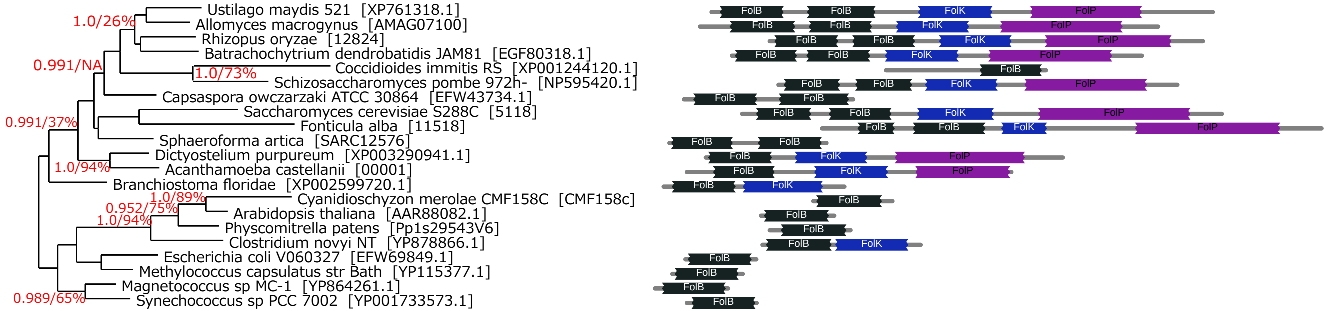

Maguire et al., Figure S4
